# The Growth of Polarization Domains in Ultrathin Ferroelectric Films Seeded by the Tip of an Atomic Force Microscope

**DOI:** 10.1186/s11671-022-03688-2

**Published:** 2022-05-12

**Authors:** Mohammad Zamani-Alavijeh, Timothy A. Morgan, Andrian V. Kuchuk, Gregory J. Salamo

**Affiliations:** 1 Physics Department, University of Arkansas, Nanoscience Building, 731 West Dickson, Fayetteville, AR USA; 2 Institute for Nanoscience and Engineering, University of Arkansas, Fayetteville, AR USA

**Keywords:** Polarization domains, Applied electric field by AFM tip, Merz’s law

## Abstract

Piezoresponse force microscopy is used to study the velocity of the polarization domain wall in ultrathin ferroelectric barium titanate (BTO) films grown on strontium titanate (STO) substrates by molecular beam epitaxy. The electric field due to the cone of the atomic force microscope tip is demonstrated as the dominant electric field for domain expansion in thin films at lateral distances greater than about one tip diameter away from the tip. The velocity of the domain wall under the applied electric field by the tip in BTO for thin films (less than 40 nm) followed an expanding process given by Merz’s law. The material constants in a fit of the data to Merz’s law for very thin films are reported as about 4.2 KV/cm for the activation field, $E_{\mathrm{a}}$, and 0.05 nm/s for the limiting velocity, $v_{\infty }$. These material constants showed a dependence on the level of strain in the films, but no fundamental dependence on thickness.

**Supplementary Information:**

The online version contains supplementary material available at 10.1186/s11671-022-03688-2.

## Introduction

**Fig. 1:**
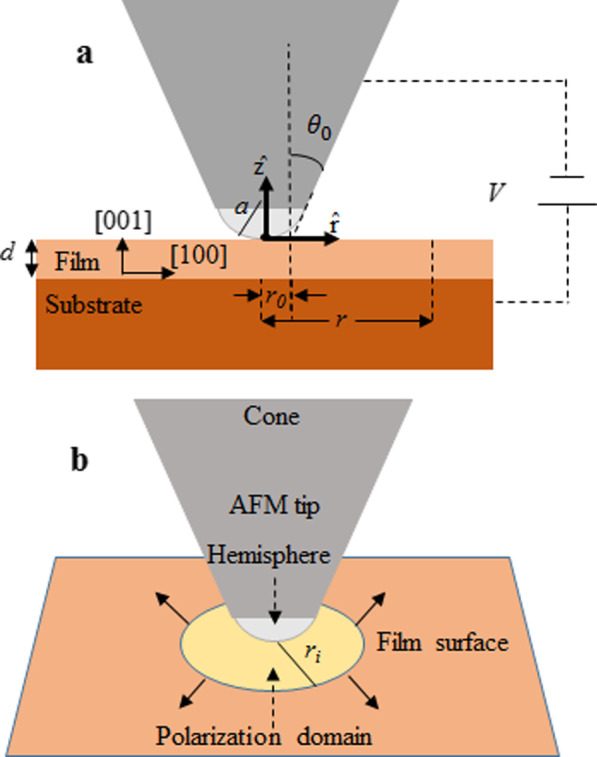
**a** For the conductive AFM tip-ferroelectric film-conductive substrate, the origin is at the tip–surface contact point; *V* is the applied voltage; *a*, the tip apex radius; *r*, is the lateral distance from the origin; $r_{0}$, the distance between the intercept of the cone with the surface and the origin; *d*, the thickness of the film; and $\theta _0$, the cone half angle. [001] is the c-direction of the film and [100] the a-direction. **b** Polarization domain expands over time under the electric field applied by the AFM tip. $r_{i}$ is about a tip diameter away from the origin and where the electric field due to the cone dominates

There have been several recent studies of ferroelectric polarization domain formation under an applied electric field [1–15], many of which have focused on the application of PFM to both form and probe polarization domains [6–16]. In the PFM technique, the tip of an atomic force microscope (AFM) makes contact with a thin film at a specific point and applies an electric potential across a thin film using the AFM tip as one electrode and the back side of the sample as the second electrode (Fig. 1a). As a result, an electric field is applied to the film in a region that is defined by the tip geometry at the point of contact. The electric field in the region just beneath the tip is nearly perpendicular to the thin film surface, except for fringing electric field effects. Under the force of the applied electric field, the ferroelectric dipoles align in a direction dependent on the field direction and crystal orientation, resulting in the formation of aligned dipoles directly under the AFM tip [6–15]. This is observed using the PFM to scan over the thin film which measures and maps out the polarization perpendicular to the surface. However, the ferroelectric dipoles are not only observed to quickly align perpendicular to the surface directly below the hemisphere of the tip, but also slowly expand laterally to a region beyond several tip diameters [6–15] (Fig. 1b). The expansion is observed to depend on the magnitude of the electric field [6–8, 14, 16], temperature [1, 6, 17, 18], and importantly, defects and strain in the material [19, 20]. This expanding region of dipole alignment perpendicular to the surface is referred to as a polarization domain, the process of aligning the dipoles as poling and its dynamic expanding edge as a domain wall. Several explanations for the observed expanding polarization domain in thin films have focused on treating the AFM tip as a point charge and argued a dependence on thin-film thickness [6–15]. Here we give a totally different explanation based on: (1) the electric field produced by the potential due to the cone geometry of the AFM tip as opposed to a point charge at the AFM tip and (2) that the speed of the expanding domain is fundamentally independent of the thickness of ultrathin ferroelectric films.

The velocity of the lateral expansion of this domain wall is predicted by [8–11, 19, 21]:

1 $$\begin{aligned} v=v_{\infty }\exp \left( -\frac{U}{kT}\left( \frac{E_\mathrm{c}}{E}\right) \,^\mu \right) \end{aligned}$$

In this equation, *v* is the velocity of the lateral expansion of the domain wall; *E*, the applied electric field; $v_{\infty }$, the limiting velocity for an infinite applied electric field; *U*, the energy barrier between the initial and final polarization; $E_\mathrm{c}$, the critical electric field; *k*, the Boltzmann constant; *T*, the sample temperature; and $\mu$, an exponent factor [19, 20, 22, 23]. In the case that the exponent $\mu$ is equal to 1, the equation for the domain wall velocity reduces to an expression referred to as Merz’s law [1]:

2 $$\begin{aligned} v=v_{\infty }\exp \left( -\frac{E_{\mathrm{a}}}{E}\right) \end{aligned}$$

where $E_{\mathrm{a}}$ is called the activation field and is equal to $(UE_\mathrm{c})/(kT)$. Physically, the phenomena of the expanding polarization domain caused by the AFM tip can be understood as due to the decrease in the magnitude of the applied electric field perpendicular to the film, as a function of the lateral distance from the tip, and the corresponding electric field-dependent probability per unit time of aligning ferroelectric dipoles. Since the electric field is lower, further from the tip, the probability of aligning ferroelectric dipoles is lower, and it consequently takes a longer time to align dipoles, resulting in a progressively expanding domain region and domain wall.

While this behavior, expressed in Eqs. 1 and 2, has been investigated [6–15, 24, 25] by several investigators, the role of the thickness of the ferroelectric thin films is not yet clear. This is evidenced by the fact that very different models for the lateral spatial dependence of the electric field perpendicular to the surface, away from the AFM tip (Fig. 1a), have been proposed to describe the lateral expansion of the domain wall, and these models vary on the role of the film thickness [6–15, 26, 27]. In this paper, our objectives are to: (1) demonstrate that the electric field in an ultrathin film due to an AFM tip, at a lateral distance of only one tip diameter away from the tip (Fig. 1b), in a direction perpendicular to the surface, is produced by the cone of the tip rather than produced by the hemisphere of the tip; (2) uncover the dependence of the lateral expansion of the polarization domain on film thickness and electric field; and (3) determine the corresponding material constants, $\mu$, $E_{\mathrm{a}}$ and $v_{\infty }$ for BTO thin films.

## Experimental Method

### Applied Electric Field in Thin Film by AFM Tip

The experimental setup used to study domain formation and its lateral expansion, and an AFM tip are depicted in Fig. 1a.

Using this experimental setup, several different analytic approximations have been developed for the electric field in the film perpendicular to the surface. For example, in one case, the spherical section of the AFM tip is treated as an effective point charge and used to find the electric field in the film at all lateral distances away from the tip [7, 8, 12, 26, 27]. Other researchers [10, 13, 14] have used the electric field of the AFM tip to be given by a point charge only for $r>>a$ as:

3 $$\begin{aligned} E_{z}(r)=\frac{Va}{rd} \end{aligned}$$

In Eq. 3, *V* is the applied voltage; *a*, the tip apex radius; *r*, the lateral distance from the tip–surface contact point; and *d*, the thickness of the film. In general, analytic approximations for the electric field in the film due to the AFM tip have been preferred over exact numerical simulations because they can be immediately used to verify Eqs. 1 or 2 by comparing directly with experimental observations for the observed velocity of the domain wall. In this paper, we define the geometry of the tip as composed of two parts: a hemisphere which is attached to a truncated cone (Fig. 1). Consequently, we also assume that the electric field due to the tip in the thin film, perpendicular to the surface, $E_{z}^\mathrm{film}$ , can be modeled as the electric field due to the tip hemisphere, $E_{z}^\mathrm{tip}$, plus the electric field due to the truncated cone, $E_{z}^\mathrm{cone}$.

4 $$\begin{aligned} E_{z}^\mathrm{film}=E_{z}^\mathrm{tip}+E_{z}^\mathrm{cone} \end{aligned}$$

It is important to note, however, that all published expressions for the electric field in the thin film neglect the electric field due to the cone section of the tip, when examining the velocity of the domain wall [6–15]. This is a reasonable assumption for films that are thicker than the tip radius and for distances very close to the tip where the fringing field of the tip hemisphere is greater than the electric field of the cone. However, for thin films, the perpendicular component of the field due to the hemisphere is very small at distances on the order of one tip diameter away from the tip. In this case, the electric field at the film due to the cone becomes dominant and must be considered. In this paper, we only consider the velocity of the domain wall at lateral distances greater than about one tip diameter away from the tip for which $E_{z}^\mathrm{film}\approx E_{z}^\mathrm{cone}$ applies. While the current investigation is focused on the expansion of the polarization domain in BTO, it is important to also consider the domain expansion as a good test for an accurate expression for the electric field due to an AFM tip. When the AFM tip is used to study the electromechanical response of materials, a good understanding of the expression for the electric field produced by the tip can be critical [28, 29]. To find an analytic expression for the electric field due to the cone in the film, we used the Laplace equation in the spherical coordinate system with electric field boundary conditions. The details of finding the electric field due to the cone are geometrical and are given in Additional file 1. The analytic expression for the component of the electric field due to the cone, perpendicular to the film surface, is given by:

5 $$\begin{aligned} E_{z}(r)=\frac{V}{(r-r_{0})\epsilon _{c}\mid \ln \mid \tan {\frac{\theta _0}{2}}\mid \mid } \qquad r>r_{i} \end{aligned}$$

In Eq. 5, $\epsilon _{c}$ is the dielectric constant of the BTO film in the c-direction; *r*, the lateral distance from the origin; $r_{0}$, the cone intercept with the surface from the origin; *V*, the applied voltage; $\theta _{0}$, the cone half angle of the tip; and $r_{i}$, the radius of the domain before domain expansion is dominated by the cone electric field (Fig. 1). In our model, we considered the AFM tip specification as measurement parameters, and when different tips are used, the tip parameters ($r_{0}$, $\theta _{0}$) should be known and substituted in Eq. 5 to arrive at the same values of activation field and limiting velocity. Therefore, we used a reference sample designed for calibrating AFM tips (test grating tips (TGT1) made by ScanSens), to determine the tip parameters: $r_{0}$, $\theta _{0}$ and *a* (see Fig. 1 and Additional file 1).

### Measurement

The experimental measurements were taken on ferroelectric BTO ultrathin films (2, 10 and 40 nm), grown by a Riber 32 MBE, on strontium titanate doped with niobium (STO$(0.05\%$ Nb)) (purchased from CrysTech) using the shutter-RHEED method [30]. The growth temperature was $650\,^{\circ }$C, and barium and titanium cells were operated at $590\,^{\circ }$C and $1830\,^{\circ }$C, respectively. RHEED oscillations indicate the thickness of the films to be 2, 10 and 40 nm and are given in Additional file 1 (Additional file 1: Fig. S3). Each RHEED oscillation indicates the added growth of one BTO monolayer, which corresponds to a thickness of 0.4 nm. The root mean square (RMS) of surface roughness $(R_{q})$ of the films measured by AFM were 0.16, 0.19 and 0.24 nm for 2, 10 and 40 nm films, respectively, which corresponds to the order of one monolayer of BTO, and XRD 2 theta–omega shows a single-crystal structure for BTO films (See Additional file 1; Additional file 1: Fig. S4 and Additional file 1: Fig. S5) .

**Fig. 2:**
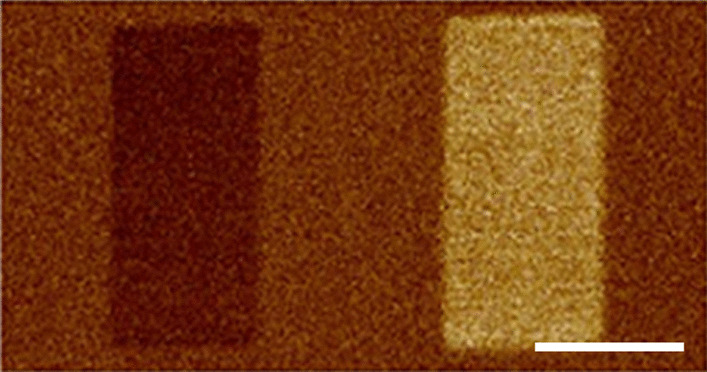
The downward [00-1] domain on left is formed by scanning − 4 V, and upward [001] domain on right is formed by scanning $+$ 4 V. The white line on the figure is 1 $\upmu$m

BTO is one the most investigated ferroelectric materials and is utilized in many applications including capacitors [31], electro-optical and electromechanical devices [32], dynamic random access memories [33] and among others due to its excellent dielectric, ferroelectric and piezoelectric properties [34, 35]. The formation of polarization domains can play a role in each. BTO can have dipoles aligned along any one of the three perpendicular crystal directions: [001], [010] or [100] [34]. The ferroelectric state of the films was aligned (poled) upward ([001] direction) or downward ([00-1] direction) by choosing the applied potential to be positive or negative. To observe the polarization direction, we measured the vertical component of polarization (Fig. 2) using an AFM D3100 Nanoscope V with PFM tips (SCM-PIT-V2) both made by Bruker. The specification of the AFM tips given by the manufacturer was confirmed using our measurements on the reference sample (TGT1). These data are given in Additional file 1.

**Fig. 3:**
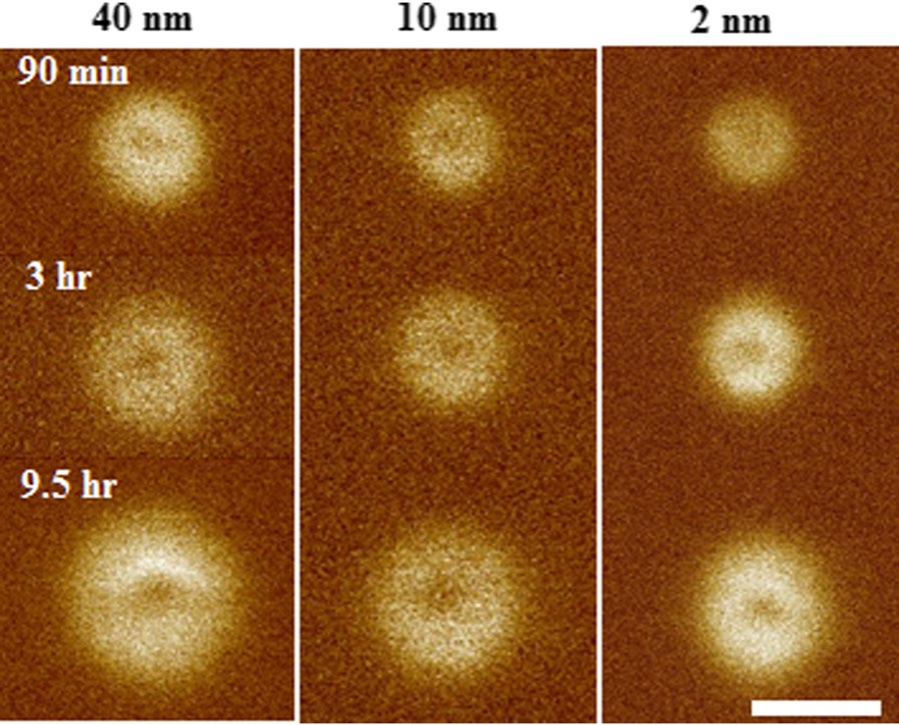
Polarization domain of [001] poled domains on 2, 10 and 40 nm BTO films as a function of time after poling with 7 V by tip $\#1$. The white line on the figure is 400 nm

At the start of each experiment, we prepared our sample with dipoles aligned in the downward direction [00-1] or opposite to the growth direction, forming a micron-size poled region (Fig. 2). After preparing the initial state, the tip was placed only at one specific spatial point in the poled region (as opposed to scanning when preparing the sample), for a given time *t*. As a result, ferroelectric dipoles began flipping as soon as the tip made contact with the surface and continued aligning in the [001] direction for a time *t* with the tip always fixed at the same specific point (Fig. 1b). This was followed by examining the change in the poled region of the film by PFM, scanning with a $V_{ac}$ of 1 V, at a frequency of 26 KHz. Repeating the same measurements for different times *t* indicated that a large, circularly symmetric, polarization domain was formed, with dipoles aligned along the [001] direction, and increased in diameter as a function of time for all three film thicknesses (Fig. 3). While the induced polarization is probed here conveniently using the same AFM tip, any method to probe the polarization could be used. To create the domains, the same AFM tip was used in each measurement for accuracy and comparison between different samples. During the measurement, the tip size was also periodically measured by using the reference sample (TGT1) to assure no noticeable tip deformation had taken place over the course of the measurements. In addition, a minimal contact force (0.05 V deflection set point) was used during scanning to help reduce tip deformation and the influence of stress induced by the tip on the polarization domain. Before each measurement, the sample is preheated to 200${^\circ }$C to reduce the possibility of any water content at the surface, which has been noted to affect ferroelectric domain formation [36–41]. Measurements were always taken after thermal equilibrium at room temperature was established to avoid thermal drift between the sample and AFM tip, and all measurements were taken at room temperature (68${^\circ }$F within 1${^\circ }$F). A constant low flow of dry nitrogen was also used around the sample in the AFM to minimize surface contamination. We found each of these to be important conditions to obtain reproducible results.

## Results and Discussion

**Fig. 4:**
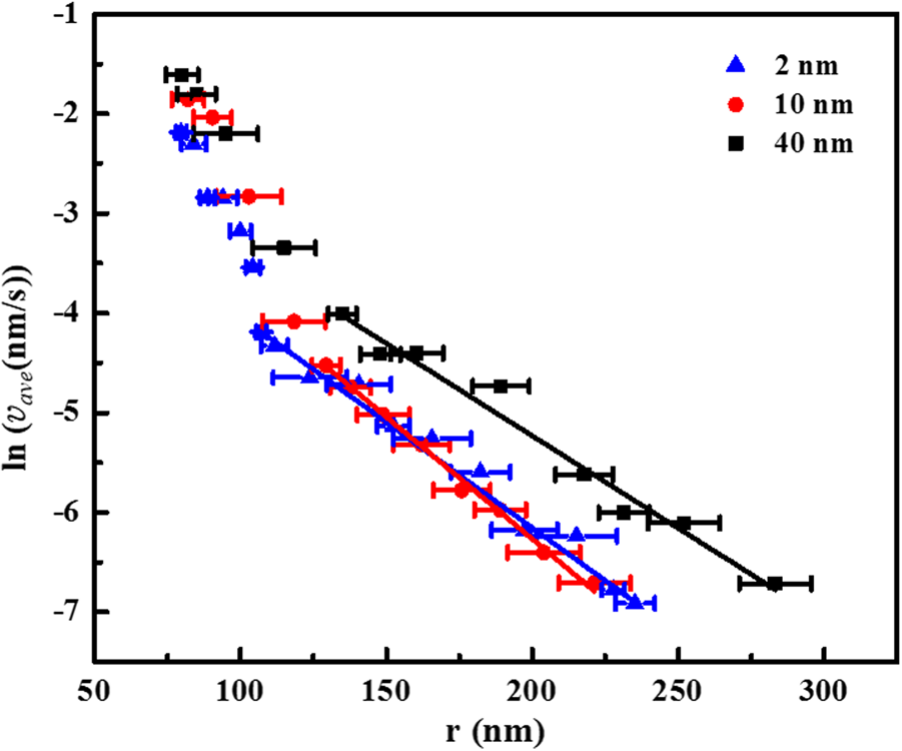
Logarithm of the average velocity of the domain wall, $v_\mathrm{ave}$ (nm/s) versus *r* (nm), the lateral distance, on 2, 10 and 40 nm films using tip $\#1$. The unit of $v_\mathrm{ave}$ is nm/s, and the unit of *r* is nm

**Fig. 5:**
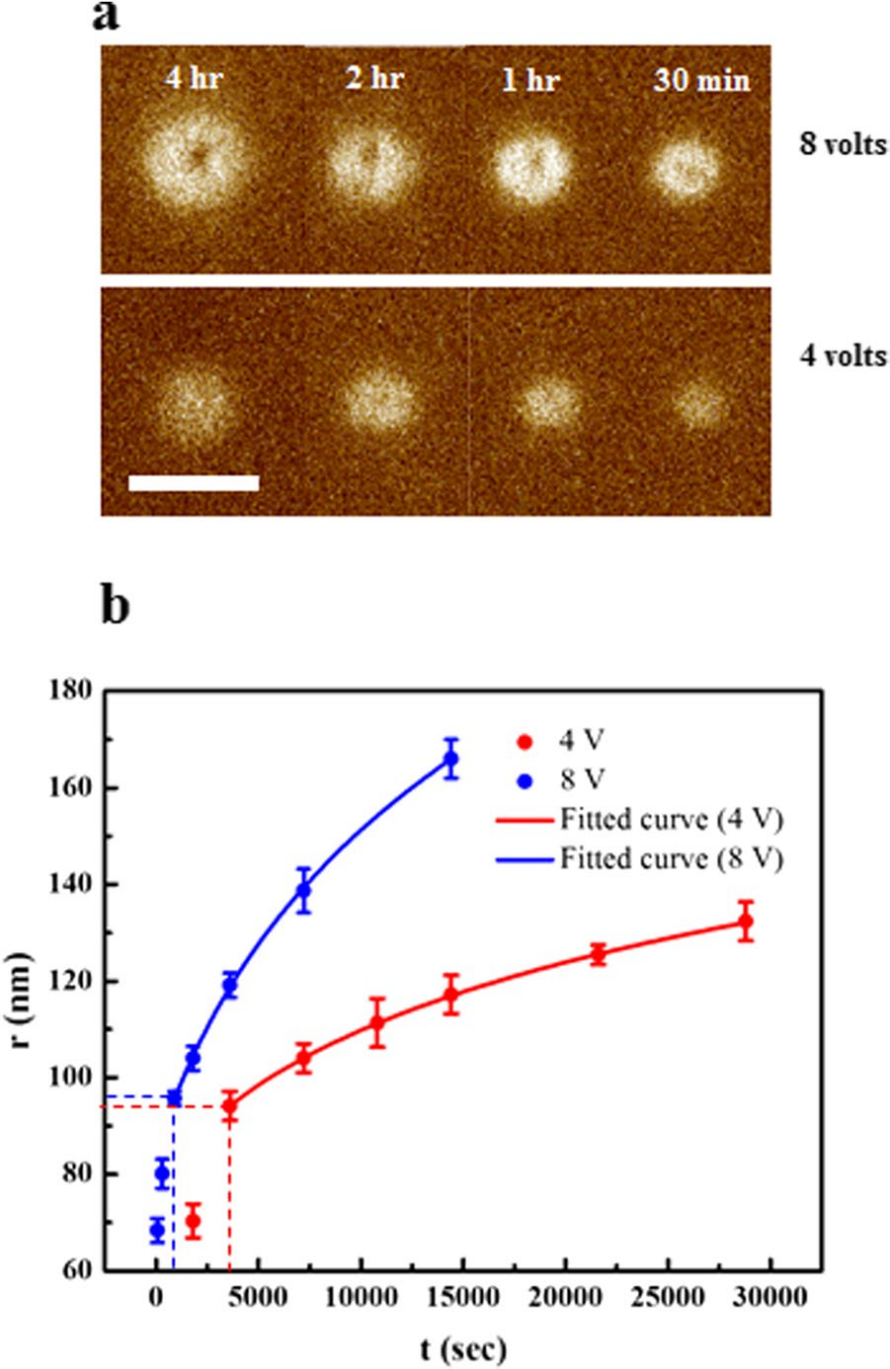
**a** Polarization domains for two applied voltages using tip$\#2$; The white line on the figure is 400 nm; **b** radius of domains, *r*, as a function of time of poling, *t* using 4 and 8 V. The data fit well to the Eq. 6 for *r* and *t* greater than $r_{i}$ and $t_{i}$

**Fig. 6:**
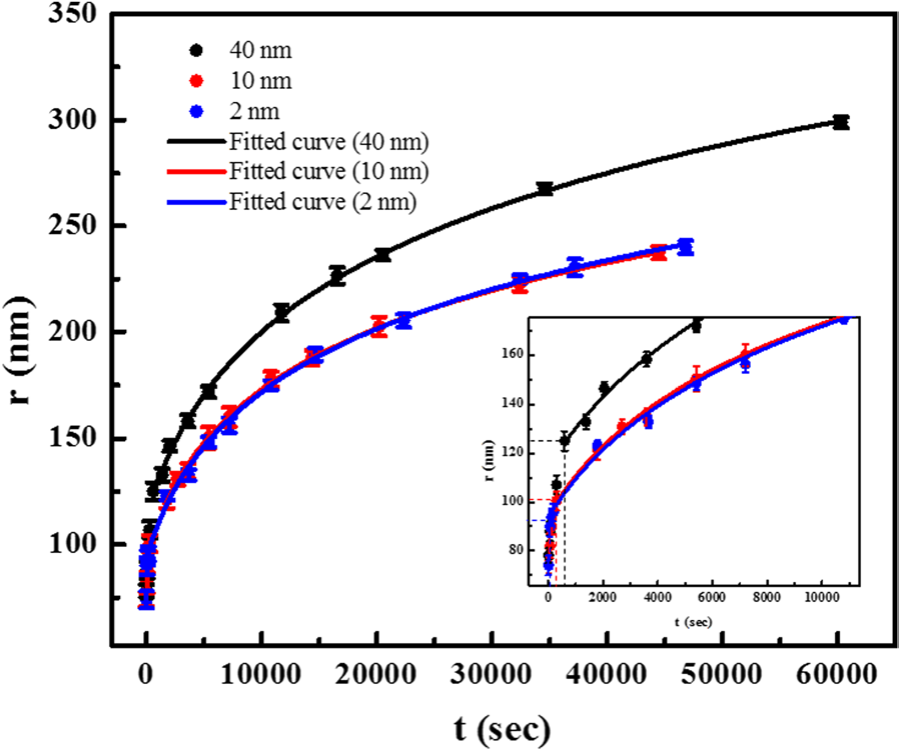
Radius of domains, *r*, vs. time of poling, *t* on 2, 10 and 40 nm films using tip$\#1$ applying 7 V. The data are accurately predicted by Eq. 6 for lateral distances greater than about one tip diameter from the tip

To study the velocity of the domain wall, we measured the position of the domain wall as a function of time, which was then used to find the constants $\mu$ , $E_{\mathrm{a}}$ and $v_{\infty }$. For example, a series of measurements for the average velocity, $v_\mathrm{ave}$, between two consecutive measured domain sizes [10, 42, 43] were used to find $\mu$ for the BTO films. To determine the domain size, the edge of the piezoelectric response of the domain is fitted to a Gaussian function followed by calculating the position for half the maximum amplitude at the domain edge. This point was then used to calculate the domain radius. The error bar is based on the error of the Gaussian fitting, which was small (Figs. 5, 6). For example, for 30 min, using 4V, the radius is 70.5±3.5 nm, while it is 104.0±2.5 nm for 8 V. The logarithm of the resulting average velocity versus the lateral distance from the tip is plotted for all the film thicknesses in Fig. 4. The data are taken with AFM tip $\#1$ by applying 7 V to form the domains for all three thicknesses (Fig. 3). By putting the electric field due to the cone (Eq. 5) in Eq. 1, the logarithm of velocity is as a function of $(r-r_{0})^\mu$. A straight line can be fitted for the region larger than about one tip diameter (120 nm) from the AFM tip indicating the exponent, $\mu$, in Eq. 1 is 1 for all the BTO films. Therefore, $\mu = 1$ indicates that for the BTO films at least, Eq. 1 reduces to Eq. 2, and Merz’s law applies.

As a result, we can apply the electric field due to the cone in Eq. 2 for the velocity of the domain wall for the domains radii larger than the tip diameter. Equation 2 for the velocity of the domain wall ($v = dr/dt$) can be integrated analytically to find *r*, the radius of the domain, as a function of time, *t*. The result for *r*(*t*) is found to be given as (details are given in Additional file 1):

6 $$\begin{aligned} \begin{aligned} r(t)&=\frac{\gamma }{E_{\mathrm{a}}}\ln \left( \frac{ E_{\mathrm{a}} v_{\infty }\exp \left( \frac{E_{\mathrm{a}}r_{0}}{\gamma }\right) }{\gamma }(t-t_{i}) +exp\left( \frac{E_{\mathrm{a}}r_{i}}{\gamma } \right) \right) \\&\gamma =\frac{V}{\epsilon _{c}\mid \ln \mid \tan {\frac{\theta _0}{2}}\mid \mid } \end{aligned} \end{aligned}$$

By comparing experimental measurements of the radius of the polarization domain as a function of time with Eq. 6, the fitting constants, the activation field, $E_{\mathrm{a}}$, and the limiting velocity $v_{\infty }$, were determined. More specifically, we determined these constants and their dependence or lack of dependence on the (a) applied electric field and (b) thickness of the BTO films.

### Dependence on Applied Electric Field

To examine the electric field dependence of Merz’s law and determine the constants, $E_{\mathrm{a}}$ and $v_{\infty }$, tip$\#2$ was used for all measurements with applied voltages of 4 and 8 V to create and investigate the expansion of polarization domains on the 10 nm BTO film (Fig. 5). To create polarization domains with voltages lower than 4 V required a very long time to form the domains. For voltages larger than 10 V, we observed electric field breakdown or too high currents. Consequently, we chose 4 and 8 V for our measurement to avoid both issues. The specification for tip$\#2$, $\theta _0$ and $r_{0}$ were measured using the reference sample, TGT1 to be 20$^\circ$ and 20 nm, respectively. $r_{i}$ and $t_{i}$ were determined for each data set to have the best fit. The data were plotted and fitted with Eq. 6 for 4 and 8 V for *r* and *t* that are greater than $r_{i}$ and $t_{i}$ as shown in Fig. 5b. The constants of the equation were found using fitting by Origin software (Table 1). The activation field was determined to be about 4.2-4.3 KV/cm and the limiting velocity was about 0.05 nm/s. As expected, the fitting constants were the same, within error bars, for both 4 V and 8 V, consistent with the fact that $E_{\mathrm{a}}$ and $v_{\infty }$ are material constants for the BTO films.

**Table 1:** Fitting constants for 4 and 8 V

*V* (V)	${\mathbf{E}}_{\mathbf{a}}$ (KV/cm)	${\mathbf{v}}_{{\boldsymbol{\infty}}}$ (nm/s)
4	4.3 ± 0.1	0.05 ± 0.01
8	4.2 ± 0.3	0.05 ± 0.01

### Dependence on the Thickness of the BTO Film

To investigate the role of film thickness, the activation field and limiting velocity were determined by comparing data to Eq. 6 for the 2, 10 and 40 nm BTO films. AFM tip$\#1$ was used on all samples to create polarization domains by applying 7 V and measuring the domain sizes at successive times. The parameters of tip$\#1$ in the fitting equation, $\theta _0$ and $r_{0}$, were measured using TGT1 and determined to be 20$^\circ$ and 45 nm, respectively. The radius of domains as a function of time of poling is plotted in Fig. 6 for three films. The data for *r* and *t* greater than $r_{i}$ and $t_{i}$ were fitted to the equation with no restrictions on the fitting constants. The analytical expression for the electric field due to the cone is dominant and in good agreement for observed polarization domain radii larger than about the tip diameter (Fig. 6). As might be expected, the constants of fitting Eq. 6 for the 10 nm film, using either tip$\#1$ and tip$\#2$, are equal within the standard deviation of fitting. In addition, since the material is the same for the 2, 10 and 40 nm films, the constants determined by fitting to Eq. 6 (Table 2) are also found to be the same. One difference exists for the 40 nm film, for which $E_{\mathrm{a}}$ = 3.2 KV/cm.

**Table 2:** Fitting constants for 2, 10 and 40 nm. *d* is the thickness of the films

*d* (nm)	${\mathbf{E}}_{\mathbf{a}}$ (KV/cm)	${\mathbf{v}}_{\boldsymbol{\infty}}$ (nm/s)
2	4.0 ± 0.2	0.05 ± 0.01
10	4.1 ± 0.1	0.05 ± 0.01
40	3.2 ± 0.1	0.05 ± 0.01

**Fig. 7:**
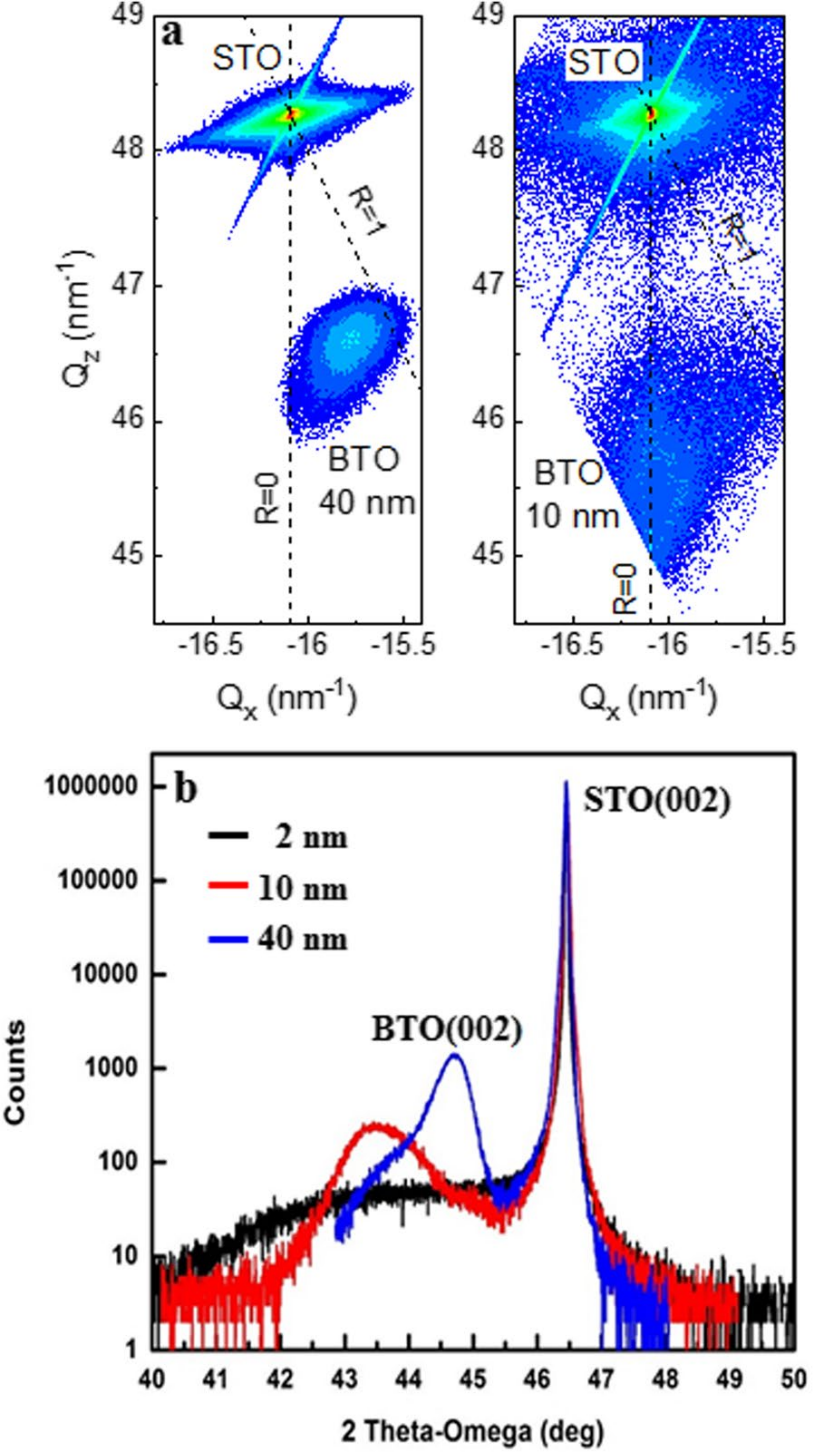
**a** Reciprocal lattice map of 10 and 40 nm BTO films on STO, **b** XRD 2 theta–omega of 2, 10 and 40 nm BTO films on STO (measured with Panalytical Materials Research Diffractrometer with Cu ka X-rays)

The XRD 2 theta–omega scan and reciprocal map show slightly different lattice parameters for 40 nm compared to the two thinner samples (Fig. 7). The 10 nm film is strained with the lattice parameters of $c=4.147\,{\AA }$ and $a=3.913\,{\AA }$. Likewise, based on the 2 theta–omega scan of the 2 nm film, this film is also strained. The strain in these two films is dictated by the substrate. However, the 40 nm film lattice parameters are $a=3.982\,{\AA }$ and $c=4.045\,{\AA }$, indicating it is nearly fully relaxed to the bulk BTO parameters [34] of a=3.992 Å$\,\hbox {and}\, c=4.036\,{\AA }$. Based on this result, the small difference in the activation field for our films can be expected due to the small difference in the compressive strain observed in the films which would tend to make it more difficult to flip the dipole direction and have greater activation field. This result is consistent with a theoretical study by Li. et al. [44] that investigated the effect of strain on the energy barrier for domain wall motion and found that the energy barrier increases by the compressive strain in the films. Since the activation field, $E_{\mathrm{a}}$, is proportional to the energy barrier, *U*, films with a larger compressive strain have a greater activation field. As a result, our observations indicate that the constants in Eq. 1 do not have a fundamental dependence on the applied voltage and thickness of the films with equal strain and that strain in the film can impact the activation field. Considering that the previous report was for 500-nm-thick films and used a different measurement technique [5], our measured values of 3 to 4 kV/cm for the activation field are in reasonable agreement with the only other previously reported (to our knowledge) value for BTO films, of 5 to12 kV/cm.

### Simulation of the Electric Field with Finite Elecment Method

**Fig. 8:**
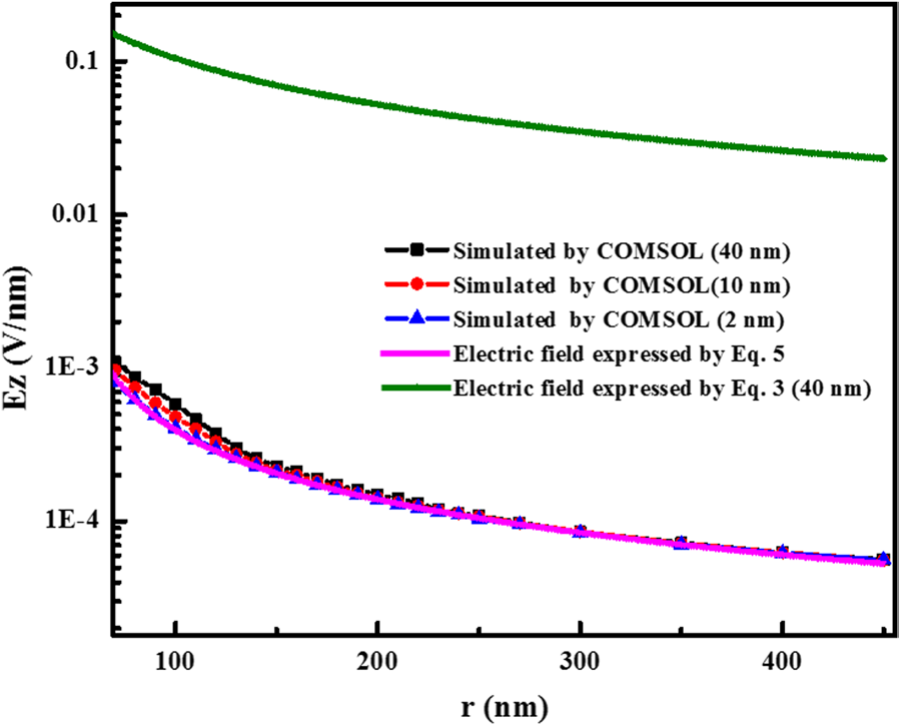
Simulated electric field applied by the AFM tip in 2, 10 and 40 nm films by COMSOL (point line), and the electric field due to the cone (Eq. 5) and the electric field expressed by Eq. 3 of previous works [10, 13, 14] (solid lines). The tip radius is 60 nm (tip $\#$1). (See Additional file 1.)

To further confirm this conclusion, a finite element method (COMSOL Multiphysics) was used to calculate the electric field in the BTO thin films between the conductive tip and substrate using the sphere–cone model for the tip. For this comparison, the same parameters for the AFM tip and for the material were used for both (1) the COMSOL simulation and (2) the corresponding analytical expressions for the electric field due to the cone (Eq. 5) and Eq. 3. They are: *V* = 7 V, *a* = 60 nm, *d* = 40 nm, $r_{0}$ = 45 nm, $\epsilon _{\mathrm{a}}=$4000, $\epsilon _{c}=$200 and $\theta _0$ = 20$^\circ$. The details of the COMSOL simulation are also given and explained in Additional file 1. The results from this comparison are shown in Fig. 8. While the electric field expressed by Eq. 3 is about two orders of magnitude larger than the simulated electric field, the cone electric field (Eq. 5) is an excellent approximation at distances $r>$130 nm for the 40 nm film, at $r>$120 nm for 10 nm film and at $r>$80 nm for 2 nm film as can be compared with experimental results (Fig. 6). Agreement is found for all three films for distances greater than approximately one AFM tip diameter. The agreement between the analytical approximation (Eq. 5) and the computational values improves with decreasing film thickness and tip apex radius.

## Conclusion

We used PFM to quantitatively study the role of film thickness and applied voltage on the expansion of the polarization domain in ultrathin films. We (1) found the electric field due to the cone of an AFM tip is needed to explain the observed behavior of the lateral expansion of the polarization domain in thin films for radii larger than about one tip diameter away from the tip; (2) developed an analytic expression for the electric field due to the cone; (3) determined the dependence of domain expansion on applied voltage and on the thickness of the film; and (4) found that PFM data taken on BTO thin films agreed with Merz’s law with exponent $\mu = 1$, limiting velocity, $v_{\infty }$ = 0.05 nm/s, and activation field, $E_{\mathrm{a}}=4.0$–4.3 KV/cm for 2 and 10 nm strained films, and 3.2 KV/cm for the 40 nm nearly relaxed film. As a result, the parameters for Merz’s law showed a dependence on strain in the film, but no fundamental dependence on thickness. The parameters $E_{\mathrm{a}}$ and $v_{\infty }$ are unique to the material.

## Supplementary Information

**Additional file 1.** Supporting information for: The growth of polarization domains in ultrathin ferroelectric films seeded by the tip of an atomic force microscope.

## Data Availability

All data are included in the manuscript and supporting information.

## Abbreviations

AFM: Atomic force microscopy
PFM: Piezoresponse force microscopy
BTO: Barium titanate
STO(Nb): Strontium titanate doped with niobium
TGT1: Test grating tips
MBE: Molecular beam epitaxy
RMS: Root mean square
