# Supplementary material for: The Growth of Polarization Domains in Ultrathin Ferroelectric Films Seeded by the Tip of an Atomic Force Microscope
Source: Nanoscale Res Lett. 2022 May 12;17:52. doi: 10.1186/s11671-022-03688-2 (PMC9098744; doi:10.1186/s11671-022-03688-2)
Supplement: Supplementary file 1 — Additional file 1. Supporting information for: The growth of polarization domains in ultrathin ferroelectric films seeded by the tip of an atomic force microscope. [file 11671_2022_3688_MOESM1_ESM.pdf]

Supporting information for: The growth of polarization domains in ultrathin ferroelectric films seeded by the tip of an atomic force microscope

M. Zamani-Alavijeh and G.J. Salamo

University of Arkansas, Physics Department, Fayetteville, AR, United States and University of Arkansas, Institute for Nanoscience and Engineering, Fayetteville, AR, United States

T. Al. Morgan and A. Kuchuk

University of Arkansas, Institute for Nanoscience and Engineering, Fayetteville, AR, United States

There are 5 sections in the supporting information: A. Development of Eq. 5, B. Specification of the AFM tips, given by the manufacturer and confirmed using AFM measurement on a reference sample (TGT1), C. Characterizations of BTO films, D. Development of Eq. 6, and E. The electric field in the film from COMSOL simulations.

A. DEVELOPMENT OF EQ. 5 FOR THE ELECTRIC FIELD IN THE FERROELECTRIC FILM.

Spherical coordinate system was used to approximate the electric field in the ferroelectric thin film between a conductive tip and a conductive substrate at lateral distances greater than about one tip diameter from the tip-surface contact (taken as origin). The geometry and the parameters of the structure were described in Fig. 1. In the approximation for the electric field in the film, the lateral size of the film and the cone is assumed to be infinite. The spherical part of the tip is neglected when we are considering lateral distances, r, greater than about one tip diameter from the origin. The film is assumed to be thin compared to the lateral distance, and the electric field in the film is also assumed to be uniform in the film. Due to the symmetry in Fig. 1, the three-dimensional Laplace equation for electric potential, ϕ, in spherical coordinate can be reduced to:

$$\nabla^2\phi = \frac{1}{r^2\sin\theta}\frac{\partial}{\partial\theta}(\sin\theta\frac{\partial\phi}{\partial\theta}) = 0 \tag{S1}$$

The solution for the differential equation is:

$$\frac{\partial\phi}{\partial\theta} = \frac{A}{\sin\theta} \qquad \phi = A\ln\mid\tan\frac{\theta}{2}\mid + B \tag{S2}$$

Where A and B are integration constants. The electric field is in the θ-direction and given by:

$$E_\theta = \frac{-1}{r}\frac{\partial\phi}{\partial\theta} = \frac{-1}{r}\frac{A}{\sin\theta} \tag{S3}$$

The electric field in the air and the film are therefore given by:

$$E_\theta^{air} = \frac{-1}{r}\frac{A_1}{\sin\theta} \tag{S4}$$

$$E_\theta^{film} = \frac{-1}{r}\frac{A_2}{\sin\theta} \tag{S5}$$

To find A_2 in Eq. S5, the electric field in the film, we make use of two boundary conditions[1]:

$$\epsilon^{air}E_\theta^{air}(\pi/2) = \epsilon^{film}E_\theta^{film}(\pi/2) \tag{S6}$$

$$-\int_{\theta_0}^{\frac{\pi}{2}} E_\theta^{air}rd\theta - \int_0^{-d} E_z^{film}dz = V \tag{S7}$$

Here, d is thickness of the film, θ_0 is the cone half angle as shown in the Fig. 1 and E_θ^{film} is approximately in the z-direction and equivalent to E_z^{film} for thin films. In the case where a thin dielectric layer forms on the surface[2–4], the change in the electric field due to the cone is negligible. Since ϵ_{film} for BTO is much greater than ϵ_{air}, we can neglect E_{film}^z in Eq. S7 and find A_2 as

$$A_2 = \frac{-V\epsilon^{air}}{\epsilon^{film}\ln|\tan\frac{\theta_0}{2}|} \tag{S8}$$

Therefore, the magnitude of the electric field due to the cone in the film is approximated as:

$$E_z^{film} = \frac{1}{r}\frac{V\epsilon^{air}}{\sin\theta\,|\ln|\tan\frac{\theta_0}{2}||\,\epsilon^{film}} \tag{S9}$$

By putting $\epsilon^{air} = 1$ and $\theta = \pi/2$, the equation for the electric field perpendicular to the film at distances greater than about one tip diameter and larger than thickness of the film is given by:

$$E_z^{film} = \frac{1}{r}\frac{V}{|\ln|\tan\frac{\theta_0}{2}||\,\epsilon^{film}} \tag{S10}$$

BTO is an anisotropic dielectric[5] with $\epsilon_c \approx 200$ and $\epsilon_a \approx 4000$. Since the electric field in the film away from the tip is mostly in z-direction, the c-dielectric constant, ϵ_c, is taken for ϵ_{film}. The cone of the tip is a truncated cone so we need to transform r to $r - r_0$ that r_0 is the distance of the intercept of the cone with the surface from the origin. By considering these substitutions Eq. S10 results in the Eq. 5 in the paper.

B. SPECIFICATION OF THE AFM TIPS, GIVEN BY THE MANUFACTURER AND CONFIRMED USING AFM MEASUREMENT ON A REFERENCE SAMPLE (TGT1).

The geometry of the tip, including the angle of the cone, θ_0, the radius of tip apex, a, as well as the intercept of the cone of the tip with the surface, r_0, are needed to find the applied electric field from the AFM tip.

To find these parameters we used a reference sample (TGT1) patterned with pointed structures having a 10 nm apex radius and 500 nm height which was used to form an AFM image using our AFM tip as shown in Fig. S1. This was then used to deconvolve out the tip parameters shown in Fig. S2 The results for "tip #1 measured" and "tip #2 measured" are compared with the "tip specifications" given by the company in Table S1.

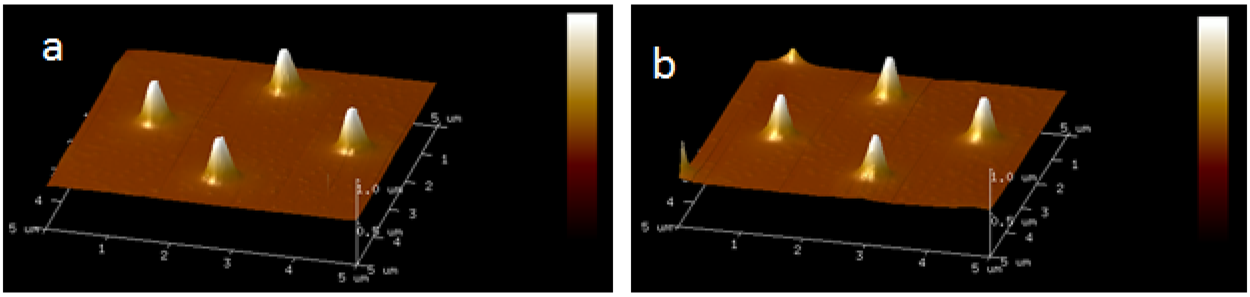

FIG. S1. 3-D AFM image of TGT1 sample with (a) tip#1, (b) tip#2. The image is 5x5 μm, the height scale is 1 μm.

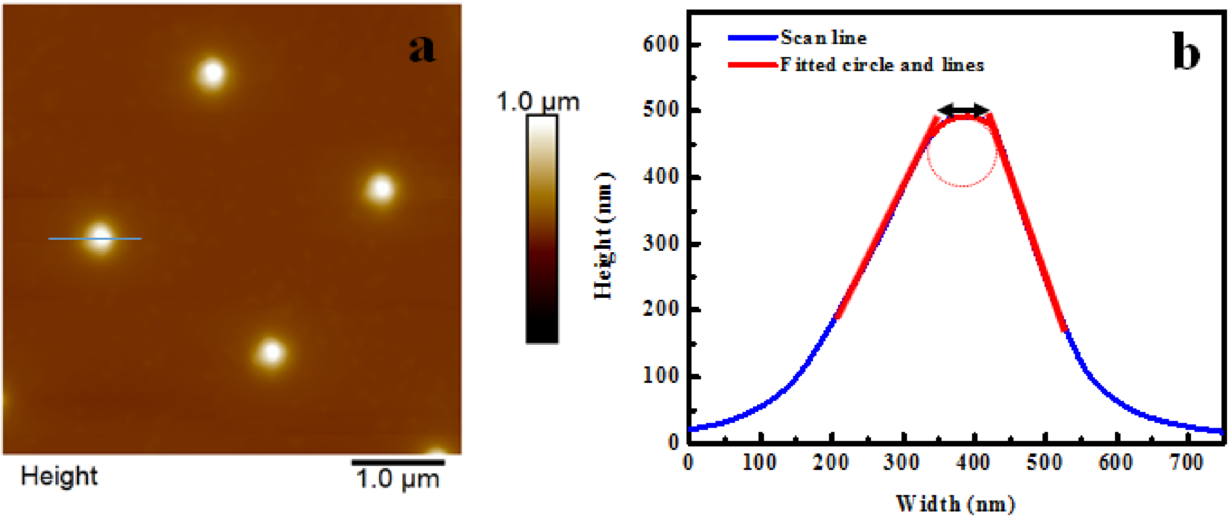

FIG. S2. (a) 2-D AFM image of TGT1 sample using tip#1, blue lines is a scan line. (b) Profile of the scan line of the AFM image, the radius of the fitted circle minus the radius of TGT1 is taken as radius of the AFM tip. Half of the double arrow length minus the radius of TGT1 is taken as r_0.

TABLE S1. Tip geometry specification and measured values by TGT1 sample.

-	a(nm)	θ_0	r_0(nm)
Tip specification	20-50	20°	-
Tip#1 measured	60	20°	45
Tip#2 measured	30	20°	20

C. CHARACTERIZATION OF BTO FILMS

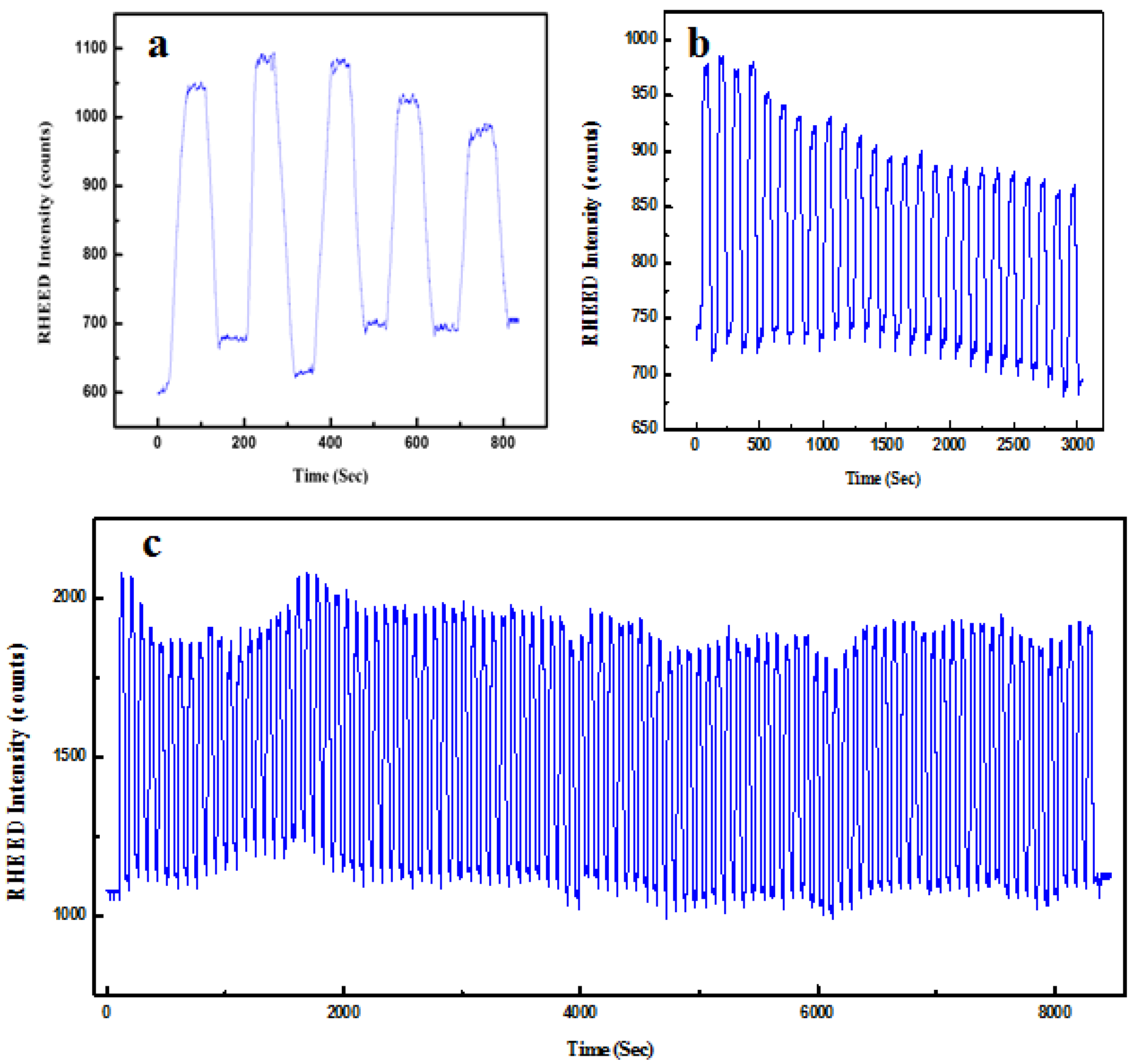

FIG. S3. RHEED oscillations of the growth of 2 nm (a), 10 nm (b) and 40 nm (c) BTO films. Each oscillation is for one BTO unit cell growth, equivalent to 0.4 nm

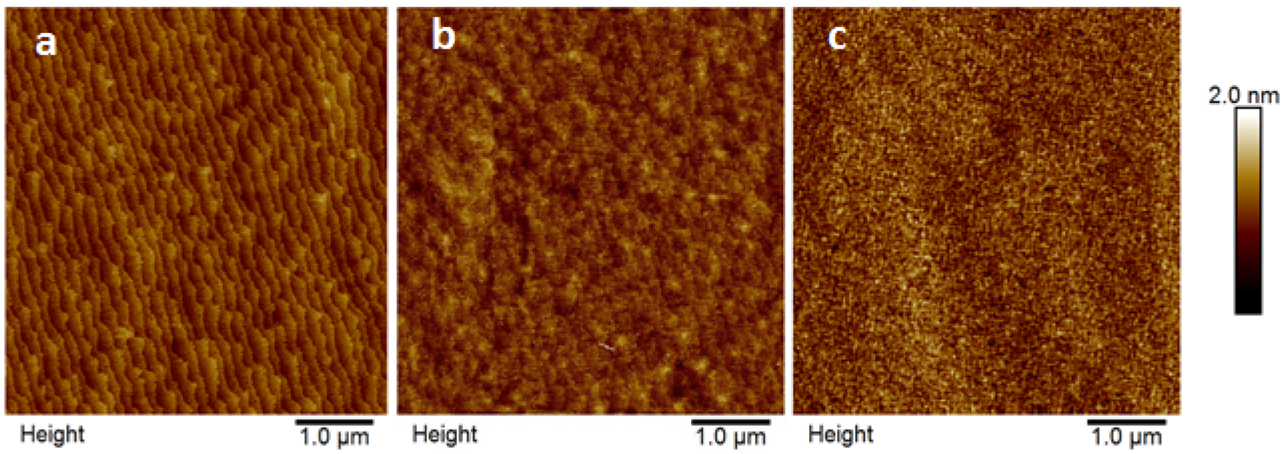

FIG. S4. AFM images of 2 nm (a), 10 nm (b) and 40 nm (c) BTO films on STO.

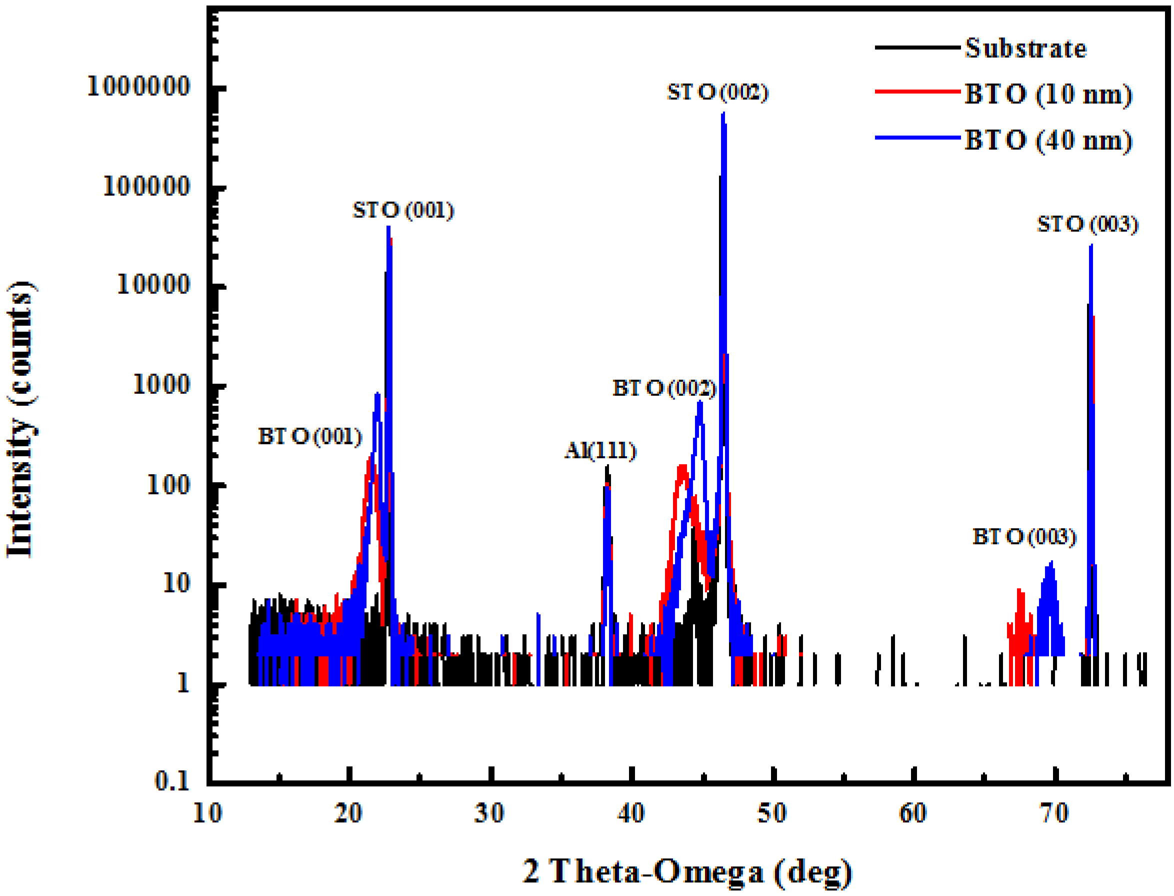

FIG. S5. 2 theta-omega XRD of STO substrate , and 10 nm and 40 nm BTO films on STO. Al (111) is from the stage of the measuring apparatus.

D. DEVELOPMENT OF EQ. 6

To find the equation that describes the domain radius as a function of the poling time, the electric field (Eq. 5) is substituted into Merz's law (Eq. 2), and the velocity of domain wall is written as dr/dt in which r is the domain radius and t the poling time. Therefore, we have:

$$\frac{dr}{dt} = v_\infty \exp\left(-\frac{|\ln|\tan\frac{\theta_0}{2}||\,\epsilon_c E_a}{V}(r - r_0)\right) \tag{S11}$$

To simplify the calculation we define γ as:

$$\gamma = \frac{V}{|\ln|\tan\frac{\theta_0}{2}||\,\epsilon_c} \tag{S12}$$

Separating r and t terms on the two sides of equation (S11) gives:

$$\exp(-\frac{E_a}{\gamma}r_0)\exp(\frac{E_a}{\gamma}r)dr = v_\infty dt \tag{S13}$$

Now we can integrate both sides with the initial values of (t_i, r_i) and final values of (t, r), and the result is:

$$\frac{\gamma}{E_a}\exp(-\frac{E_a}{\gamma}r_0)\left(\exp(\frac{E_a}{\gamma}r) - \exp(\frac{E_a}{\gamma}r_i)\right) = v_\infty(t - t_i) \tag{S14}$$

By rearranging Eq. S14 to find r as a function of t, we have Eq. 6.

E. THE ELECTRIC FIELD IN THE FILM FROM COMSOL SIMULATIONS

We used the finite element method of COMSOL Multiphysics to simulate the electric field due to the AFM tip. The AFM tip is defined as a sphere and a truncated cone (Fig. 6S). In the simulation, the cone height is 800nm, the half angle of the cone, 20°, the width of the film and substrate, 1400 nm, the dielectric constants of the barium titanate (BTO) film[5], $\epsilon_x = 4000, \epsilon_y = 4000, \epsilon_z = 200$ and the dielectric constant of the material around the tip, 1. The tip and substrate were taken as "terminals" with the voltage of 0 V and 7 V respectively. Since there are reports[2–4]on the presence of an ultrathin dielectric surface layer (carbon compounds) on ferroelectric materials, 0.5 nm dielectric layer with the dielectric constant of 2 was considered on the films. We simulated the electric field for 2, 10, and 40 nm films and two different tip apex radii of 30 nm and 60 nm. The mesh was selected based on no change in simulation with selection of a finer mesh size.

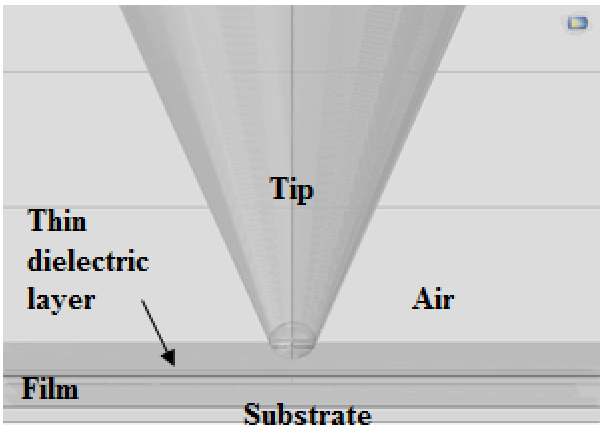

FIG. S6. Defined elements in COMSOL simulation.

The simulation shows that the electric field in the z-direction under the tip is larger near the surface of the film ($z = 0$) and decreasing moving toward the substrate ($z = -10$ nm). This is observed in Fig. S7 by comparing the slope of the electric potential vs. z at $z = 0$, to the slope at $z = -10$ nm, for a 10 nm film at $r = 0$ and $r = 10$ nm. However, the electric field becomes uniform for larger r as shown by the simulated data at $r = 100$ nm for a 10 nm film in Fig. S7.

The electric field in the z-direction at the surface of the films ($z = 0$) for different films are compared in Fig. S8 indicating the behavior of the electric field as a function of lateral distance, r, for different thicknesses of the film. The electric field is clearly larger for thinner films than thicker films at $r = 0$. However, this order changes at $r = 50$ nm and the electric field becomes bigger for thicker films due to the greater fringing field from the tip hemisphere compared to thinner films. At larger distances, however, the electric field in thicker films approaches the electric field of thinner film, becoming thickness independent. As discussed in the paper, the COMSOL simulation was used to further support using Eq. 5, the electric field due to the cone, for the electric field at lateral distances larger than about tip diameter.

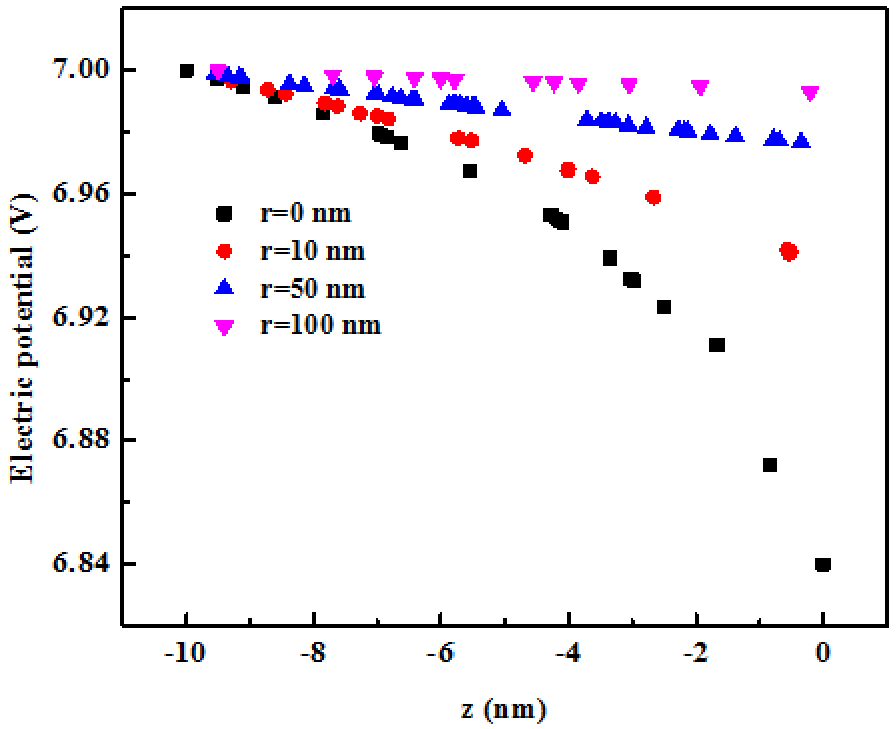

FIG. S7. Electric potential vs. z for the 10 nm film at different radial distances ($r = 0$, $r = 10$ nm, $r = 50$ nm and $r = 100$ nm) using tip#1.

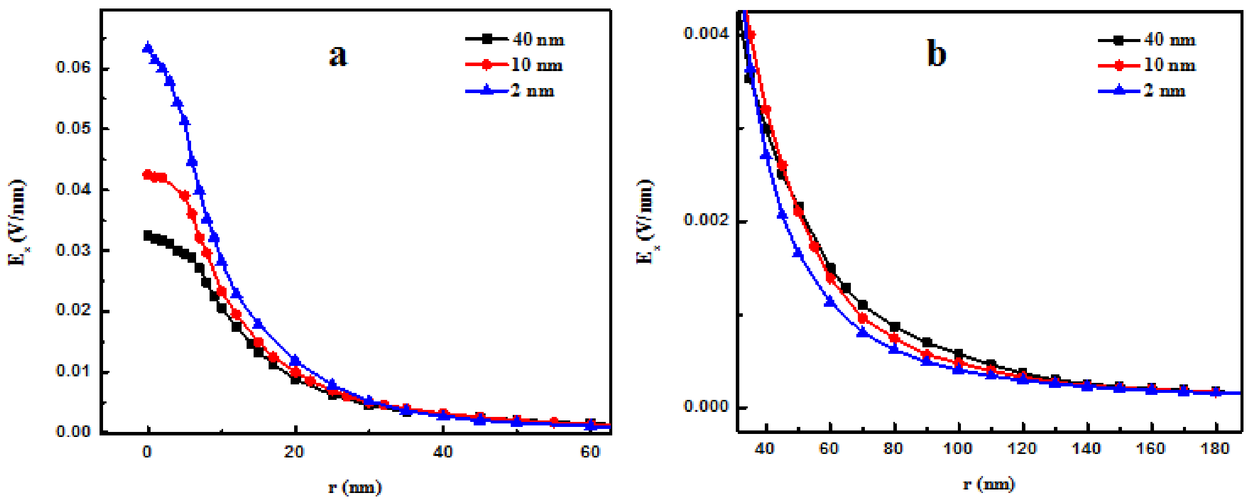

FIG. S8. Simulated vertical electric field at the surface of the films induced by AFM tip#1 in 2,10, and 40 nm films vs. lateral distance from the tip, r for (a) 0 nm$< r <$ 60 nm, (b) 40 nm $< r <$ 180 nm.

[1] Wang B, Woo C. Atomic force microscopy-induced electric field in ferroelectric thin films [Journal Article]. Journal of Applied Physics. 2003;94(6):4053–4059.

[2] Tomashpolski Y, Lubnin E, Sevostianov M. Auger-spectroscopy of surface-layers in ferroelectrics [Journal Article]. Ferroelectrics. 1978;22(1-2):785–786.

[3] Baniecki J, Ishii M, Kurihara K, Yamanaka K, Yano T, Shinozaki K, et al. Chemisorption of water and carbon dioxide on nanostructured $BaTiO_3$-$SrTiO_3$(001) surfaces [Journal Article]. Journal of Applied Physics. 2009;106(5).

[4] Peter F, Szot K, Waser R, Reichenberg B, Tiedke S, Szade J. Piezoresponse in the light of surface adsorbates: Relevance of defined surface conditions for perovskite materials [Journal Article]. Applied Physics Letters. 2004;85(14):2896–2898.

[5] Jona F, Shirane G. Ferroelectric crystals. New York: Dover Publications; 1993.
